# Horseback Riding

**Published:** 1881-06

**Authors:** 


HALL'S
### JOURNAL OF HEALTH
AND
MISCELLANY.

E. H. GIBBS, A. M., M. D. - - EDITOR.

Founded in 1854, and published Monthly without Interruption since that Time.

### HORSEBACK RIDING.

EXERCISE is essential to health. Upon its proper use depends the health and usefulness of every individual. But in its npplication extremes must be avoided. Long continued quiet brings on an enfeebled condition of the body; and over-exertion, long continued, wears it out. One is rust, the other wear. The advantages of exercise as a preventive of disease, or as a curative agent, are frequently not only lost but become positively injurious from want of knowledge or judgment in applying it.

Aimless exertion is less beneficial than that where the mind—the ambition—is satisfied. Let a man swing a sledge hammer many times each day, striking only an empty anvil, and the labor will do him positive injury, but if the blows fall on hot iron which is being shaped into horseshoes or other useful articles, the mind and body are both brought into healthful and harmonious action; there is an object to be accomplished, and such labor becomes invigorating and healthful. There are two methods of exercise quite distinct from each other in their effects. One is ACTIVE, the other PASSIVE. Then there is the combination of both, which is best illustrated in HORSEBACK RIDING, the best and most exhilarating of all the methods.

In all the affairs of life, whether of business or pleasure, there are certain "drawbacks." If expenses and losses exceed the income, there comes a time when the business must stop. In mental or bodily labor, as in disease, there is a constant waste of muscle and brain which, when carried beyond the recuperative power of the system, results in death. Active exercise, with its waste of tissue, is essential to health, and is the most important curative agent known, in nearly all diseases, when carried just far enough to enable nature to replace the waste matter with better material. In this way we often get rid of diseases without medicine.

But there is a class of invalids who cannot endure active exercise. The recuperative power is not sufficient to supply the waste caused by great exertion. Mechanical means have been devised to meet these cases. The *energy* is supplied by a steam engine, so that a person may be vigorously "shaken up" without effort on his part. I have no doubt great benefit is often derived by these means. For example, a person has an arm paralyzed; the hand is fastened to a handle on the end of a rod that rapidly vibrates back and forth, just far enough to turn the arm half around and back again. Another rather amusing, but still useful, device is a sofa with perhaps twenty holes through it, up through which the padded ends of pieces of wood project alternately when the machinery is in motion. The patient lies upon this sofa, and then slowly rolls over. In a few minutes every portion of the body receives a thorough punching. This is *passive* exercise. I believe that a plan that combines both of these methods is the best. Horseback riding, under proper conditions and restrictions, combines these plans. It may not meet all cases. If that were so, it would be an exception to all human agencies and devices.

I will refer briefly to the objects to be attained by this plan of exercise, and the reasons why they are more likely to be attained by this than by any other. The class of patients that most frequently require this treatment are those suffering from lung troubles, in various stages. In these cases there is a want of vital power and energy, and active exercise alone, without sufficient recuperative force, would increase the trouble. What is required, is to give the system a thorough "shaking up;" for this the horse supplies the *energy*. The fatigue of the patient is passive, induced without effort on his part. The motion compels him to expand the lungs, by which more oxygen is received, and thus the blood becomes rapidly purified, and enabled to resist or throw off disease.

Not the least of the advantages of horseback riding is its mental effect. There are some things that are generally regarded as mere matters of detail, to which I attach great importance. An invalid is usually sensitive and capricious, and disturbed or distressed by many things that a well person would not notice. However harsh and cruel a man may become toward his fellow men, he seldom loses his love for fine horses. In selecting a horse for an invalid, I should choose one that is sound, handsome, gentle and affectionate; a strong firiendship is soon established between the patient and his horse, greatly to the advantage of the patient, whose thoughts have a new occupation. Men who have the care of horses and cattle usually enjoy good health, and invalids often seem to have been restored to health by devoting their time to the care of horses. For an invalid, always select a horse that trots. The gallop is of little use. If possible, there should be ostensibly some other object besides health in horseback riding. It would be desirable to have some business, as an agency for a commercial house, that would require the patient to travel on horseback a few miles every day, not only for the purpose of paying expenses, but to make him feel that he is mingling in business affairs, and is not out of "the battle of life;" for nothing affects an invalid so unfavorably as to have his friends relieve him from the cares of business.

If one's means would admit of it, he could purchase two horses and saddles, and invite a relative or friend—who should be in good health—to travel with him through the pine forests of North Carolina, with a view of studying the resources of the State; this makes it a journey of business; but he should not take a sick man for a companion. "Two of a trade never agree," or rather they would agree so well that they would be likely to converse more about their own ailments than about business. The travelers should adopt the "Bill of Fare" of the people—pork and hoe-cake—and avoid close sleeping rooms. The nearer the approach to "roughing it," the greater the benefit to be expected, the object being to get the greatest amount of exercise possible, short of great fatigue, in a pure atmosphere that is perfumed by the dense forests of pine. I believe that this treatment would, in a few months, restore to health every case of incipient lung trouble, and perhaps a majority of those who have advanced to the second stage of the disease.

